# Nivolumab‐induced acute tubular injury: A case report

**DOI:** 10.1002/ccr3.6991

**Published:** 2023-03-07

**Authors:** Hui‐Hsin Yang, Chia‐Wen Chang, Tai‐Di Chen

**Affiliations:** ^1^ Department of Pharmacy Chang Gung Memorial Hospital, Taoyuan Taoyuan City Taiwan; ^2^ Department of Nephrology Chang Gung Memorial Hospital, Linkou Taiwan

**Keywords:** acute tubular injury, lymphocyte transformation test, Nivolumab

## Abstract

Nivolumab belongs to immune checkpoint inhibitors (ICIs). ICIs‐induced kidney injury is rare and acute interstitial nephritis (AIN) is the majority. A 58‐year‐old woman had gastric cancer treated with nivolumab. Her serum creatinine (Cr) increased to 5.94 mg/dL post 2 cycles of nivolumab and co‐administered with acemetacin. A kidney biopsy showed acute tubular injury (ATI). Nivolumab rechallenge was done and Cr worsened again. The lymphocyte transformation test (LTT) indicated a strong positive for nivolumab. Although rare, ATI due to ICIs could not be ruled out, and LTT is a tool to identify the culprit.

## Graphical abstract

Clinical course and management of Nivolumab‐induced ATI.

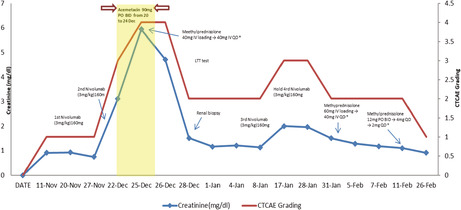

## 1 BACKGROUND

Nivolumab belongs to ICIs. Among many immune‐related adverse events (IRAEs) due to ICIs, IRAE nephritis is around 2–5%.^1^ Kidney biopsies of ICIs‐induced nephritis show that the most common histological feature is AIN. Once IRAEs develop, corticosteroids are the drug of choice.

## 2 CASE PRESENTATION

A 58‐year‐old postmenopausal woman was diagnosed with right breast cancer (invasive ductal carcinoma), ER:(3+, 90%), PR:(3+, 2%), HER‐2: [1+], KI‐67:15% (Luminal B), and stage I (T1cN0M0) in May 2016. She received partial mastectomy and Sentinel node excision on June 26, 2016. She kept on adjuvant hormone therapy with Tamoxifen after operation. Synchronous stage IV gastric adenocarcinoma with ovarian metastasis and peritoneal carcinomatosis (T3N2M1) was diagnosed in July 2016. Systemic chemotherapy with XELOX and FOLFORI was administrated until September 2017. Ovarian metastatic tumor progression and bone metastasis were noted in September 2017. Laparotomy with bilateral salpingo‐oophorectomy and partial omentectomy was performed on October 17, 2017. Palliative systemic therapy with Nivolumab (3 mg/kg) was initiated on November 24, 2017. The second cycle was given on December 11, 2017, and her Cr level was 0.91 mg/dL, urinalysis showed negative for protein and hemoglobin before nivolumab initiation and Cr level was 0.74 mg/dL on December 27 before the second cycle of immunotherapy.

**FIGURE 1:**
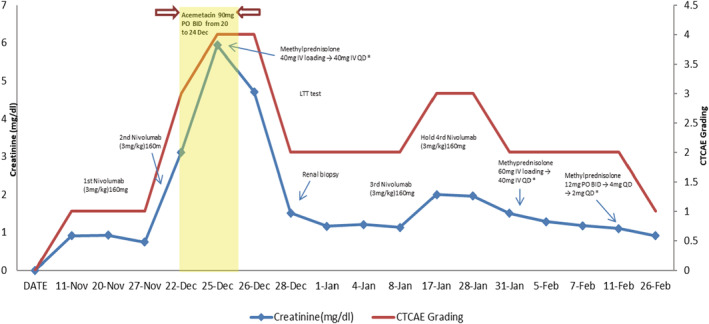
Clinical course and serum creatinine change after the initiation of Nivolumab therapy. CTCAE, Common Terminology Criteria for Adverse Events. *Methylprednisolone was applied for suspected IRAE nephritis.

The patient was found to have acute kidney injury (AKI) with a Cr level of 3.11 mg/dL (CTCAE grading 3) on December 22 through prechemotherapy lab survey, after 2 cycles of nivolumab (Figure 1). Other biochemical panels revealed sodium 141 mEq/L, potassium 5.1 mEq/L, blood urea nitrogen (BUN) 57.9 mg/dL, total protein 7.1 g/dL, and albumin 4.09 g/dL. Urinalysis showed 3+ (300 mg/dL) for protein and 3+ for hemoglobin. On urine microscopic examination, there were 306/μL red blood cells and 33/μL white blood cells. We also performed serologies tests (immunofixation electrophoresis, monoclonal free light chain, Antinuclear Antibody, Anti‐dsDNA, C3, C4, hepatitis B surface antigen, hepatitis C virus antibody, Protein electrophoresis) related to autoimmune diseases to ruled out other possibilities. After hospitalization, intravenous fluid was applied under the impression of dehydration. Obstructive uropathy was also suspected, and urologist was consulted. Follow up lab survey on December 25 showed AKI in progression. The Cr level elevated to 5.94 mg/dL (CTCAE grading 4) and BUN was 83.9 mg/dL. Before nivolumab usage, the kidney echo revealed bilateral moderate hydronephrosis with obstruction, status post bilateral double‐J catheters insertion on 2017/10/20. Intravenous pyelography showed right upper ureteral angulation and stricture. Left hydronephroureter with an obstructive level at L4‐5 due to compression or adhesion by left ovarian vein.

IRAE in kidney was suspected in the presence of proteinuria and hematuria. We exclude the cause of AKI was postrenal insufficiency with underlying glomerulonephritis. No other IRAEs develop relative to nivolumab.

Methylprednisolone (1 mg/kg/day) was applied for suspected IRAE nephritis on December 25, 2017 (Figure 1). After medication, urinalysis showed negative for protein and 2+ for hemoglobin. On microscopic examination, there were 9 red blood cells and 9 white blood cells. Meanwhile, LTT was conducted on the next day but the results were only available after one month while renal biopsy was performed on December 28. The histological finding of renal biopsy showed ATI. No crescent, segmental sclerosis, necrosis, nor thrombus was found.

Interstitium shows focal and mild inflammation composed of mononuclear cells. Tubulointerstitial fibrosis is less than 5%. The Immunofluorescence study showed negative stains for IgG, IgA, IgM, C3, C1q, and kappa and lambda light chains.

**FIGURE 2:**
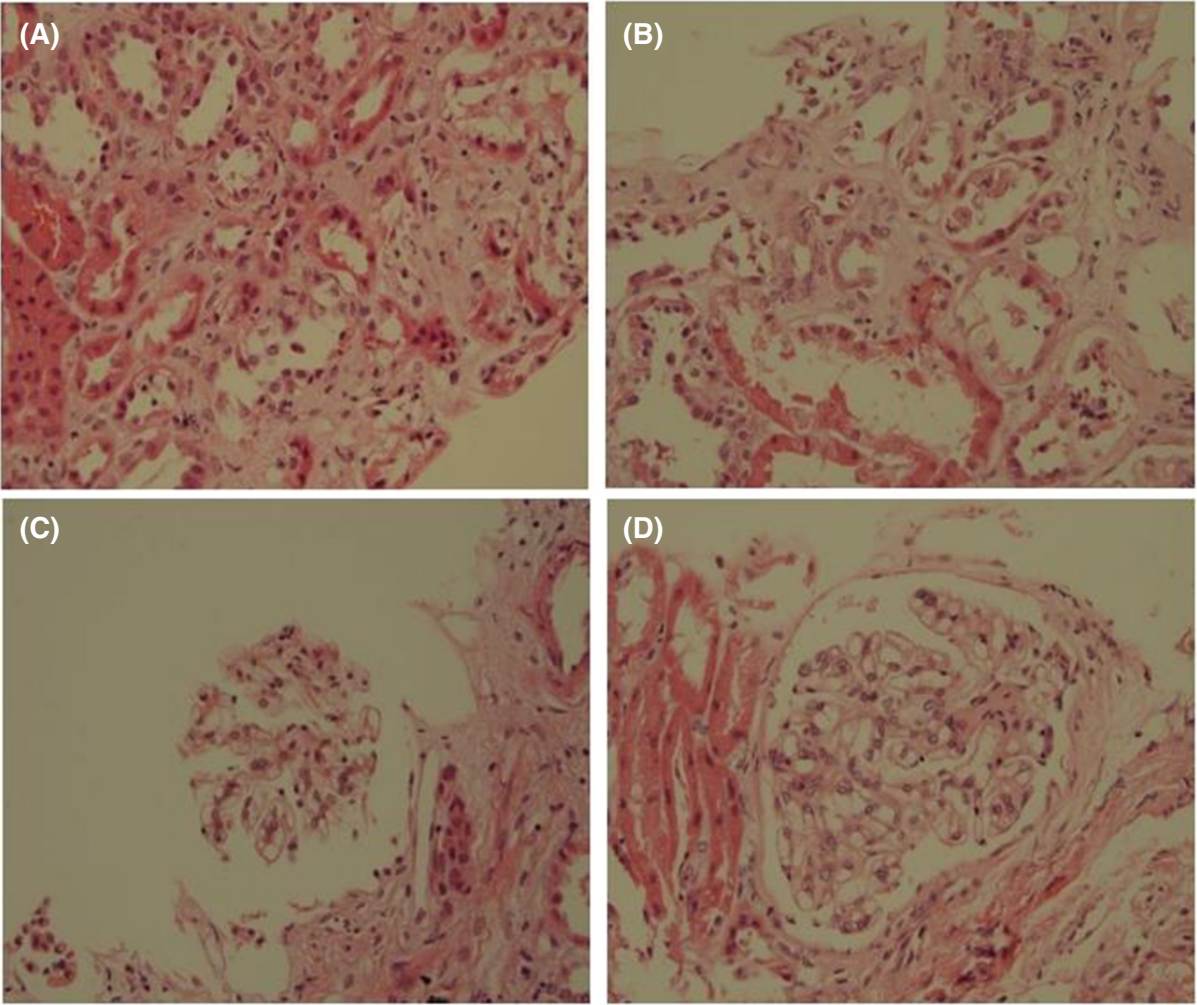
Kidney biopsy showed acute tubular injury. Tubules showed anisonucleosis, attenuation of tubular cells, and lumen dilatation. There is no tubulointerstitial nephritis. (A, B) Glomeruli are unremarkable, and there is no immune complex‐mediated glomerulonephritis nor thrombotic microangiopathy. (C, D) There is no tubulointerstitial nephritis in those images.

Reported findings in IRAE nephritis such as tubulointerstitial nephritis, immune complex glomerulonephritis, or thrombotic microangiopathy are not seen (Figure 2). One month later, LTT showed a strong positive for nivolumab (Table 1).

**TABLE 1:** Lymphocyte transformation test.

Category	LTT test value			
	GNLY fold change	Granzyme B fold change		
			Reference value	
Nivolumab	17.6	>Max	+/−	(1–1.5)
Ipilimumab	1.32	1.6	+	(1.5–2)
Pembrolizumab	2.93	105.37	++	^2–3^
PHA	3.83	2.53	+++	(>3)

*Note*: The lymphocyte transformation test (LTT) indicated a strong positive for nivolumab.

Traced the patient's medication use, She had not received PPIs during this episode but she had prescribed acemetacin (Non‐Steroidal Anti‐Inflammatory Drug, NSAID) from 2017 12/20–24 for pain control, which may contribute to her renal impairment as the Cr level showed an increase (Figure 1). After discussing with the nephrologist, NSAIDs‐related acute tubular nephropathy was preferred.

We discontinued acemetacin on December 24, 2017, and keep methylprednisolone 60 mg once daily from December 28, 2017 to January 2, 2018. The patient's renal function improved rapidly. Cr level was 1.16 mg/dL (CTCAE grading 2) and BUN was 31.3 mg/dL on January 1, 2018. We discontinued methylprednisolone on January 2, 2018. Third cycle of immunotherapy with Nivolumab was re‐initiated on January 4, 2018. The patient denied any discomfort after immunotherapy and she was discharged on January 8, 2018.

At fourth cycle immunotherapy on January 18, 2018, impaired kidney function was noticed again. The Cr level was 2.0 mg/dL (CTCAE grading 3) on January 17 and BUN was 44.6 mg/dL. She denied urine output decreased, dysuria, or back pain.

Besides, interval progression of the right hydronephrosis despite double‐J in place was noted. We consulted Urologist. for progression of the hydronephrosis. Ureteroscopy and change bilateral double‐J tube were performed. We hold the 4th immunotherapy and readmitted the patient for further treatment. Therefore, methylprednisolone 60 mg QD was prescribed from February 1, 2018, to February 7, 2018. The patient's renal function improved gradually (Figure 1). The Cr level returned to 1.17 mg/dL (CTCAE grading 2) on February 7, 2018, and we tapered methylprednisolone to 40 mg/day.

Under stable condition, the patient was discharged on February 12, 2018. She received methylprednisolone 4 mg per day at our outpatient department. Her renal function remained stationary under treatment.

## 3 DISCUSSION

We encountered a case of ATI induced by nivolumab for the treatment of gastric cancer. ICIs are a new category of immunotherapy against several malignancies. Various organ systems can be affected by IRAEs. Although the incidence of ICI‐ related AKI is relatively rare (2–5%) compared to other organs.^2^ An increased number of reports on ICI‐induced kidney injuries has been observed in recent years. Cortazar et al reported that the overall incidence of AKI was 2.2% among a total of 3695 patients treated with ICI monotherapy.^3^

The types of nephropathies caused by the ICI class vary enormously even when induced by a single agent such as nivolumab. Cortazar et al.^3^ described the histologic features of ICI‐associated nephrotoxicity in patients who receive kidney biopsy. AIN was the most common pathologic lesion encountered in their report.^3^ Similarly, Shirali et al.^4^ described the histologic features of ICI‐induced AKI in 6 lung cancer patients who all had AIN. Omar et al reported 16 cases of nephrotoxicity linked to ICI therapy, and the most common pathological finding is acute tubular necrosis 1. In addition, AIN can frequently co‐occur with other forms of glomerular pathologies injury including Minimal Change Disease, membranous nephropathy, IgA nephropathy, pauci‐immune glomerulonephritis, and lupus‐like glomerulonephritis.^5^, ^6^, ^7^ AIN pattern on kidney biopsies seems to be the predominant histological character of renal complications in IRAEs. However, our case proved to be ATI. The clinical manifestation of AIN is similar to ATI, but AIN has a more hidden onset while ATI arises from direct tubular epithelial injury with rapid deterioration of renal function.^8^, ^19^

The median time from ICI start to AKI is variable. In the patient of our case, the time relapsed from the initial exposure to nivolumab plus longer half‐life acemetacin until the onset of AKI was 30 days. This delayed response is in line with the observations of a case series of 13 patients with ICI‐induced AKI, in which the median time from initiation of an ICI to AKI was 91 days (range: 21–245 days).^3^, ^9^, ^10^, ^11^ The heterogeneous and delayed onset of AKI implies that the mechanistic pathway is distinct from the typical drug‐induced hypersensitivity reaction. ICIs‐induced AIN may be due to “reprogramming” of the immune system, leading to the loss of tolerance against endogenous kidney antigens, as opposed to a delayed‐type hypersensitivity reaction.^3^, ^12^, ^13^ The proposed mechanisms are the concomitant drug (NSAID) exposure, iatrogenic and xenobiotic molecules can trigger an immune response either by itself or after binding to tubular antigens, thus acting as haptens. These T cells primed by drugs administration became latent over the time, however, by ICIs they can be reactivated leading to loss of tolerance. This might be why some of the cases had a long latency period time before AIN occurred.

Our patient had received short‐acting NSAIDs without causing abnormal renal function before this incident. The use of long‐acting acemetacin during nivolumab therapy may result in increased nephrotoxicity according to the longer half‐lives. After our patient had experienced a second treatment of nivolumab (3 mg/kg) 160 mg and acemetacin 90 mg, the serum Cr surged from 0.91 to 5.94 mg/dL compared to nivolumab alone (Naranjo score = 6). According to the severity of nephrotoxicity provided by KDIGO guidelines, our patient belongs to stage 3 AKI (serum Cr: 3.0 times baseline, or ≥4.0 mg/dL, or urine output: <0.3 mL/kg/h for ≥24 h, or anuria for ≥12 h).^14^ It is highly suspected that exposure to NSAIDs is a risk factor that may be associated with AKI. We strongly speculate that programmed death‐1 inhibitor (Anti‐PD‐1) therapy may have disrupted long‐standing tolerance of NSAIDs by modulating peripheral immune tolerance for the drugs that had been safely used previously in our patient, resulting in clinically significant nephrotoxicity.

As we know, anti‐PD‐1 therapy may drive an autoimmune variant of interstitial nephritis, similar to the induction of autoimmune diabetes, possibly mediated by the loss of peripheral tolerance to self‐reactive T cells.^12^, ^15^ The disruption of PD‐1 signaling might also break tolerance to drug‐specific effector T cells that are critical to the pathogenesis of AIN.^12^, ^16^ In this scenario, anti‐PD‐1 therapy reactivates exhausted drug‐specific T cells primed by exposure to nephritogenic drugs, including PPIs (proton pump inhibitors) and NSAIDs but subsequently inhibited by PD‐1 signaling. Either scenario resulted in increased effector T‐cell migration and function, leading to clinically significant renal injury. This hypothesis is based on Cortazar et al. report that 14 out of 19 acute tubulointerstitial nephritis patients^3^ while using ICIs, combined suspected drugs such as PPIs and NSAIDs. Medications such as PPIs, NSAIDs, and antibiotics are an important cause of AIN on their own and have been very commonly reported as concomitant drugs in patients who develop ICI‐related AIN resulting in shorter progression‐free survival and overall survival.^17^ Among patients diagnosed with ICI‐related kidney injury, 69% were provided combination therapy, including antibiotics (9%), NSAIDs (22%), or PPIs (54%).^18^, ^19^ Therefore, we believe that the patient's ICI‐induced AKI is highly related to the combinational use of other medications and that the presentation of AKI may not be AIN but other possible manifestations.

Our therapeutic approach included the discontinuation of the possible offending agent (nivolumab, acemetacin) and daily administration of systemic corticosteroids for IRAEs according to guidelines. After 5 days of treatment, serum Cr dropped. Current guidelines20 for ICI‐induced AKI are formulated by the Society for Immunotherapy of Cancer per Common Terminology Criteria for Adverse Events (CTCAE version 5.0).^20^ For persistent grade 2 (doubling of Cr or higher), the guidelines recommend discontinuing ICIs. They also recommend a corticosteroid taper that begins when Cr improves to grade 1 or below.^2^ The guidelines from the National Comprehensive Cancer Network specify a starting dose and duration for the corticosteroid taper: 0.5–1 mg/kg/day for grade 2 and 1–2 mg/kg/day for grade 3 AKI with the dose being tapered over 4–6 weeks after Cr decreases to less than or equal to grade.^12^, ^21^ In the scenario of ICI‐induced AKI, there are currently rechallenge guidelines to come after. For grade1‐2, renal IRAEs upon resolution to ≤ grade 1 consider resuming concomitant with steroid if Cr is stable and permanent discontinuation of immunotherapy is warranted in the setting of severe (grade 3–4) proteinuria.

In the study from Cortazar et al, only 22% of patients with stage 2 or 3 AKI were rechallenged; only 23% of rechallenged patients occurred in recurrent ICI‐induced AKI; of those with recurrent AKI, all but one responded to the second course of glucocorticoids.^18^, ^22^, ^23^ The latency period for ICI‐related AKI after retreatment was shorter than during first use, all but 1 patient achieved complete or partial recovery of renal function after rechallenge. In contrast, Meraz‐Munoz et al.^24^ have reported a 12.5% recurrence rate.

In our case, after the third cycle of nivolumab in the condition without NSAIDs involved, renal function relapse occurred, giving a Cr level of 2.0 mg/dL. (Naranjo score = 7) In comparison, the second cycle of nivolumab given with acemetacin showed a Cr level of 5.94 mg/dL. Therefore, we highly suspect that acemetacin might play a role in causing AKI.

However, our case report has several limitations. First, LTT for acemetacin was not performed before nivolumab therapy. Thus, it is not known whether nivolumab therapy altered the patient's immunological tolerance against acemetacin. LTT results for suspicious drugs are required to clarify the pathophysiology of AKI. Second, the LTT detection time for nivolumab takes 3 weeks, and IHC staining is not promptly in progress. Therefore, it might delay a better understanding of the pathophysiology and etiology behind the renal disease associated with ICIs. Third, we do lack baseline urinalysis before the use of ICI and, therefore, cannot completely exclude the presence of underlying renal pathologies prior to ICI use. Four, Renal biopsy was performed not promptly after high‐dose steroid therapy and decreased creatinine to near baseline level in this case. Thus, tubulointerstitial inflammation might already disappear at the time of the biopsy, Finally, considering the half‐life of nivolumab in the human body (12 to 20 days) and the decline in renal function appears to be related to the dose and duration of exposure to NSAID, it is uncertain the causal relationship between ICI and concomitant medications known to cause ATN.

In summary, acemetacin (an NSAID)‐induced ATI was highly suspected initially. However, according to the LTT and nivolumab rechallenge results, we strongly speculated that nivolumab (an ICI) may contribute to ATI formation. From the literature review, cases of ICIs‐induced ATI had been found as well1. We should give scrupulous attention to nivolumab treatment in a case with co‐administered NSAIDs.

## 4 CONCLUSION

We reported a rare case of nivolumab‐induced ATI. A kidney biopsy is essential to exclude other possibilities such as interstitial nephritis or associated glomerular diseases. LTT is a useful tool to identify the possible culprit in spite of not being able to reveal the result in real time. It is important to note that, in patients with limited choice for ICI reuse, culprit medication associated with ICI‐related nephrotoxicity should be avoided.

## AUTHOR CONTRIBUTIONS

**Hui‐Hsin Yang:** Visualization; writing – original draft; writing – review and editing. **Chia‐Wen Chang:** Data curation. **Tai‐Di Chen:** Supervision.

## FUNDING INFORMATION

This research did not receive any specific grant from funding agencies in the public, commercial, or not‐for‐profit sectors.

## CONFLICT OF INTEREST STATEMENT

All authors have no financial or other conflicts of interest.

## CONSENT

Written informed consent was obtained from the patient to publish this report in accordance with the journal's patient consent policy.

## Data Availability

The grant was supported by Chang Gung Memorial Hospital.
